# Correction to “Investigating the Effect of Transdiagnostic Group Intervention on Depression, Anxiety, and Postpartum Stress in Women With Preeclampsia: A Quasi‐Experimental Study”

**DOI:** 10.1002/hsr2.72951

**Published:** 2026-08-26

Hatami Asl, Z., N. Jaberghaderi, M. Rezaei, N. Rezavand, and M. Kamravamanesh. “Investigating the Effect of Transdiagnostic Group Intervention on Depression, Anxiety, and Postpartum Stress in Women With Preeclampsia: A Quasi‐Experimental Study.” *Health Science Reports* 9, no. 6 (2026): e72608. https://doi.org/10.1002/hsr2.72608.

The name of the second author was misspelled and has been updated from Nasrin Jaber Ghaderi to Nasrin Jaberghaderi.

The online version of this article has been corrected accordingly.

We apologize for this error.

